# Upscale Design, Process Development, and Economic Analysis of Industrial Plants for Nanomagnetic Particle Production for Environmental and Biomedical Use

**DOI:** 10.3390/ma13112477

**Published:** 2020-05-29

**Authors:** Paulo A. Augusto, Teresa Castelo-Grande, Diana Vargas, Alvaro Pascual, Lorenzo Hernández, Angel M. Estevez, Domingos Barbosa

**Affiliations:** 1 Departamento de Ingeniería Química y Textil, Facultad de Ciencias Químicas, Universidad de Salamanca, Plaza de los Caídos, 1-5, 37008 Salamanca, Spain; dmariavsp@gmail.com (D.V.); alpascual@usal.es (A.P.); loherga@usal.es (L.H.); estevez@usal.es (A.M.E.); 2 LEPABE—Laboratory for Process Engineering, Environment, Biotechnology and Energy, Faculty of Engineering, University of Porto, Rua Dr. Roberto Frias, 4200-465 Porto, Portugal; castelogrande@sapo.pt (T.C.-G.); dbarbosa@fe.up.pt (D.B.)

**Keywords:** economic analysis, plant design and process engineering, nanomagnetic particles

## Abstract

Very few economical and process engineering studies have been made concerning the scale-up and implementation of nanomagnetic particle manufacturing into a full-scale plant, and determination of its viability. In this work we describe such a study for two types of industrial plants, one for manufacturing magnetic particles for applications in the environmental area, and the other for manufacturing nanomagnetic particles for applications in the biotechnology area; the two different applications are compared. The following methodology was followed: establish the manufacturing process for each application; determine the market demand of the product (magnetic nanoparticles) for both applications; determine the production capacity of each plant; engineer all the manufacturing process, determining all the process units and performing all the mass and energy balances for both plants; scale-up the main equipment; and determine the global economic impact and profitability. At the end both plants are found to be technologically and economically viable, the characteristics of the final products being, however, quite different, as well as the process engineering, economic analysis, and scale-up.

## 1. Introduction

A large amount of literature has been devoted to the research on the manufacture of magnetic nanoparticles [1,2,3,4,5]. The potential applications of magnetic nanoparticles have been identified and represent a broad window of research areas [6,7,8,9,10,11,12]. Examples of some existing magnetic nanoparticle products in the market for environmental and biotechnological applications are: 3320DX-SkySpring Nanomaterials, and 900062-SigmaAldrich. Nonetheless, the large majority of the manufacturing methods described in literature and their applications have not reached yet large-scale production. The main reason behind this failure is that usually when reaching industrial scale, most of the methods are simply not economical or technological viable. It is also important to notice that among all this literature almost no economic and technological study has been published considering the possible adaptation of the laboratorial manufacturing processes into a larger-scale production. It is, therefore, vital, at this stage of scientific and technological development, to analyze the economic and technological viability and adaptation of the proposed manufacturing processes in order to determine if and how they may be actually applied into industrial applications [13].

In this paper we present a study of the economic and technological viability of selected magnetic nanoparticle manufacturing processes for environmental and biotechnological applications. Results and differences between both are presented and analyzed. 

## 2. Materials and Methods 

The following methodology was applied: (a) choose the manufacturing process for each application; (b) proceed with a market study to determine the demand of the product (magnetic nanoparticles) for both applications; (c) determine the size of each plant; (d) analyze all the process steps, choose all the process units and perform all the mass and energy balances required in both plants; (e) detail scale-up design of the main equipment; and (f) analyze the global economic impact and profitability.

## 3. Industrial Design-Economic and Technological Viability

For a good estimation of economic and technological viability it is important to follow a determined thread-line [13]: First, the selection of the manufacturing process, considering the particular application, must be done; then a good study of the market will define the size of the plant; the size of the plant will define the required mass flows; these will then be the basis for the design of the equipment, the mass and energy balances, and the overall process flowsheet, leading to conclusions on the technological viability of the process; finally the overall economic analysis of the full industrial process is done and economic viability determined.

In this section we will follow this thread-line comparing the results obtained for the plant producing magnetic nanoparticles for environmental applications with the one for biotechnological applications.

It is important to be aware that making an overall economic estimation is always risky as it depends on many factors. However, to assure the accuracy of the estimations, we have based our calculations on well recognized and established economic analysis published in reference books such as [14,15].

### 3.1. Process Selection

The selection of the manufacturing process for the magnetic nanoparticles is dependent of their future application. In the cases under study, for biotechnological applications usually monodispersed particles are required with same shape and size, and nanosize is a requirement (so they behave superparamagnetically), for environmental applications polydispersed particles are not a drawback and large sizes are usually required for cheaper and more efficient real treatment processes [16].

As indicated in the introduction, many methods have been developed for the manufacturing of magnetic nanoparticles; these include methods like co-precipitation [17], hydrothermal [18], microemulsion [19], thermal decomposition [20], sol-gel [21], laser pyrolysis [22], etc. After a preselection based on applicability level, we have reduced the proposed methods to the ones presented in Table 1, where a comparison on their characteristics is given.

#### 3.1.1. Environmental Applications

For the applications in sight (adsorption, Fenton reaction, catalysis, etc.) [11,27] the main characteristics of the chosen process must be: easiness of synthesis; low-cost particles; easiness of processing; easiness of scale-up; production of large quantities of particles; high flow throughputs; and monodispersity is not a requirement. Considering these factors and the information on Table 1, the choice fell on the co-precipitation method. 

Among all co-precipitation methods, the inverse co-precipitation is preferred [28,29,30,31,32,33] due to its superior process control and characteristics of the obtained particles. In order to choose the best values for the process variables (drying temperature, type of alkaline base, concentration of precursor solution, type of surface agent, etc.) a laboratorial study was done and is presented in [28]. Based in this article the characteristics of the process to be used in large-scale production are: use NH_4_OH as alkaline base, no surfactant required, concentration of salt equal to 0.2 M and drying temperature of 90 °C, while other variables are as detailed in [28] with the modification of using ambient atmosphere instead of inert atmosphere so we could shorten the reaction time and increase production capability, obtaining a mixture of micron and nano-sized particles [28]. The global reaction (1) for this process is:

3FeSO_4_ + 6NH_4_OH + ½ O_2_ → Fe_3_O_4_ + 3H_2_O + 3(NH_4_)_2_SO_4_ (1)

#### 3.1.2. Biotechnological Applications

For the applications in sight (drug delivery, magnetic hyperthermia, etc.) [27,34,35] the main characteristics of the chosen process must be: easiness of synthesis; monodispersity; easiness to scale-up; production of moderate quantities; low flow-throughputs; good shape control and crystallinity and low nanosizes (particles should behave supermagnetically). Considering these factors and Table 1, the choice fell on the hydrothermal method. 

The final particles will be delivered with a carbon layer at their surface, so they can be further functionalized according to each specific need.

The method we have chosen to upscale will be the one followed by [36], choosing to use as reactants the iron salt, starch and sodium acetate, and as reaction conditions a temperature of 80 °C during 20 min and then a hydrothermal treatment of 20 h. The involved reactions (2)–(4) are [37,38,39,40]:

FeCl_2_·4H_2_O + 2CH_3_COONa → Fe(OH)_2_ + 2NaCl + 2CH_3_COOH + 2H_2_O (2)

2Fe(OH)_2_ + ½ O_2_ → 2FeOOH + H_2_O (3)

Fe(OH)_2_ + 2FeOOH → Fe_3_O_4_ + 2H_2_O (4)

Global reaction between (3) and (4):

3Fe(OH)_2_ + ½ O_2_ → Fe_3_O_4_ + 3H_2_O (5)

Global general reaction (6):

FeCl_2_·4H_2_O + 2CH_3_COONa + 2Fe(OH)_2_ + ½ O_2_ → 2NaCl + 2CH_3_COOH + Fe_3_O_4_ + 5H_2_O (6)

### 3.2. Market Study

**Figure 1:**
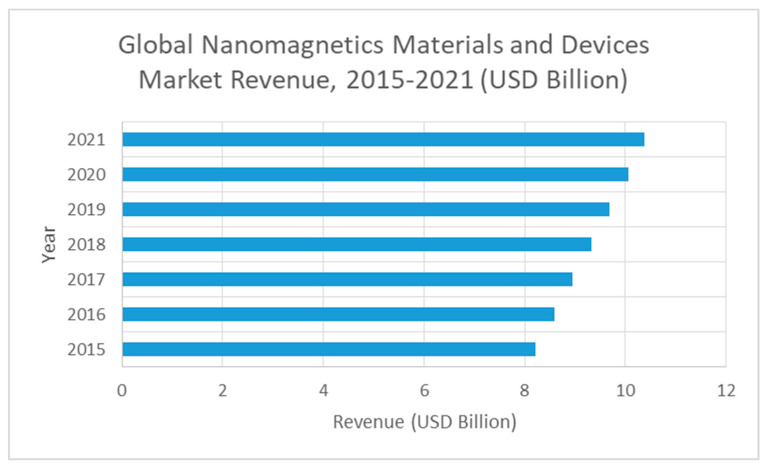
Global market for nanomaterials (based on data from [42]).

Nanomagnetic materials are a very recent commodity. Therefore, market data is very limited and still restricted. Spanish internal market is not big enough and therefore the global market must be analyzed. It is important to notice that in Europe nanomaterials and nanotechnologies are considered a key enabling technology. The global market value of products that incorporate nanotechnologies as their key component was estimated at € 700 billion in 2015 and is estimated at € 2 trillion by 2020 (generating, at the same time, between 2 and 6 million jobs, respectively) [41]. According to various reports, a large increase is expected in the global nanomaterials market, at least, until 2021, as shown in Figure 1. 

The largest difference between environmental and biotechnological applications are their market and flow quantities that are handled. In fact, environment tends to be a low-cost market with high-throughputs, while biotechnology is usually a high-cost market with low-throughputs. This marks a very distinctive difference between the demand of each market: environmental applications require simple (including microsized particles to handle large flows) and low-cost particles (no need for good shape and functionalized particles as they would represent an unbearable cost when comparing with competitive technologies), while biotechnological applications require more complicated (small nanosized particles) and functionalized and well-shaped (thus more expensive) particles.

#### 3.2.1. Environmental Applications

The majority of the current nanomagnetic particles in the market are directed towards the pharmaceutical, biotechnological and biomedical applications. Concerning environmental applications, the market share is low and almost no data is available. From the world market economy values presented in Figure 1, we may conclude that this increasing market represents a good opportunity for well-planned business plants, and not only for biotech applications. However, due to the low market share corresponding to environmental applications and to the low-level of competition we choose a conservative figure of about 0.5% of the worldwide nanomagnetic particle production for our plant production. This represents 40 million euros in annual production. The next step is to settle the price for the main product of our plant (nanomagnetic particles). By consulting the literature, we see that the price of magnetic nanoparticles varies depending on their utility, characteristics and form of presentation. For commercial nanoparticles without any functionalization, the cost can range from $380/kg (for iron oxides, both magnetite and maghemite) to $2255/kg (for nZVI) [43]. Other authors indicate that the price of 1 kg of maghemite nanoparticles varies between $200 and $400 depending, as already mentioned, on the size of the particles, the purity and the method of synthesis [44]. In Table 2 we present the current prices practiced by the company Skyspring Nanomaterials Inc. 

For our study we will consider a price of 380 €/kg, which corresponds to a production of about 100 metric ton/year.

#### 3.2.2. Biotechnological Applications

**Figure 2:**
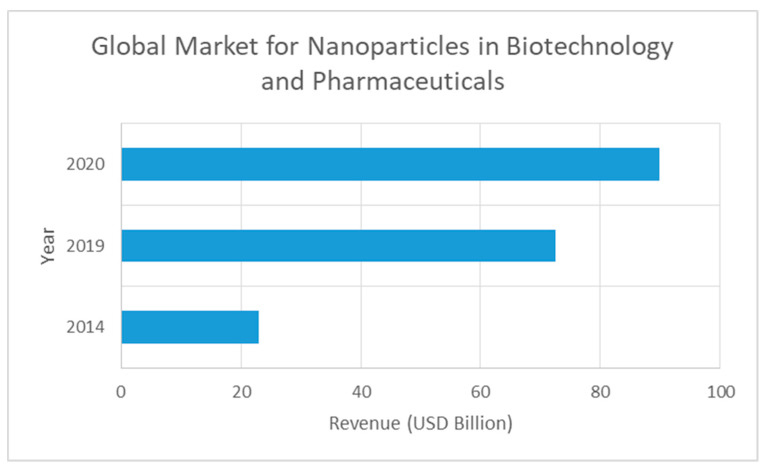
Global market for nanoparticles in biotechnology and pharmaceuticals (based on data from [46,47]).

Biotechnological applications are responsible for a large share of today’s market of nanomagnetic particles. It is a novel and open business opportunity. Nonetheless, few data exist (concerning markets) and many fierce competitors are in the market. In order to determine the market share, besides the previous detailed factors and Figure 1, it must be also taken into account the numbers indicated in Figure 2 (estimated demand of 89.84 billion US$ for 2020). Therefore, and considering the special high-level competitivity and high-value product, we consider that, as a conservative value, we will be able to supply about 0.10% (0.0915% updated to 2020 figures) of the world market with our plant. This corresponds to about 200 million euros.

To settle a price for the produced particles we analyze again Table 2 noting that in the case of our plant the particles to be produced will be delivered covered with a carbon layer (prepared for further functionalization). Therefore, we have analyzed prices of particles produced by other suppliers, like Sigma-Aldrich, and observed that particles with more or less similar properties to the ones being produced in our plant, for example, those containing PEG layer, are sold at a price of 316 €/10 mL [48]. In order to be more competitive, we will opt for a lower price for the final particles produced at our plant: 118.50 €/10 mL. Hence, a total of 6300 kg/year of nanomagnetic particles covered with carbon for biotechnological applications should be produced at the plant.

### 3.3. Size of the Plants

To estimate the size of the plant is required to balance between profits (in this case the income due to the selling of the particles) and the costs (implementation and production costs). This step only constitutes a first approach in order to determine the minimum profitable production size, as the full economical balance that is presented later in this article is only made after we have previously established all the process engineering [13]. For the calculation details the reader is addressed to the Supplementary Material.

#### 3.3.1. Environmental Applications

**Figure 3:**
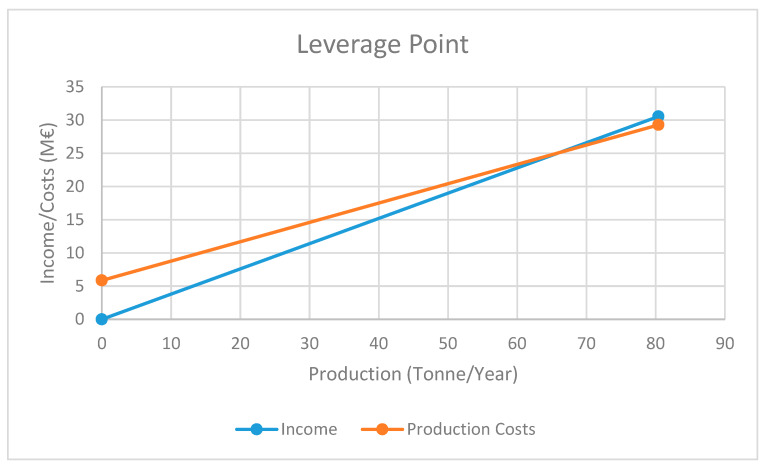
Minimum production capacity of the plant for profit (environmental applications).

Balancing between the income and the costs (details presented in Supplementary Material) we obtain the graphical depiction represented in Figure 3. As we see, the income line (Y = 0.38X, where Y are the income/costs measured in M€/year, and X the production capacity measured in tonnes/year) and the cost line (Y = 0.2914X + 5.8578, where the fixed costs were assumed to be 20% of the maximum production costs) intersect at 66.1 tonnes/year, which represents the minimum capacity value of the plant in order to get a balance between income and costs (zero profit), knowing that working at higher production capacities will always lead to positive profit values. For our plant, in this case, the maximum capacity will be 80.4 tonnes/year (the value we chose for our total production), above the minimum profitable production amount. 

#### 3.3.2. Biotechnological Applications

**Figure 4:**
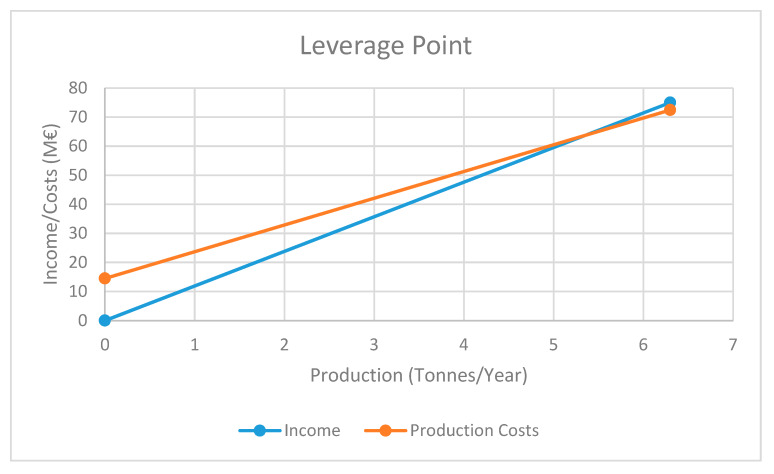
Minimum production capacity of the plant for profit (biotechnological applications).

By making the same balance between profit and costs, we obtain the curves represented in Figure 4. The intersection of the income line (Y = 11.905X) and cost line (Y = 0.1981X + 14.487) is at 5.35 tonnes/year, which represents the leverage point between Income and Costs, and represents about 85% of the total maximum production defined for our plant (6.3 tonnes/year).

### 3.4. Process Engineering and Energy and Mass Balances

**Figure 5:**
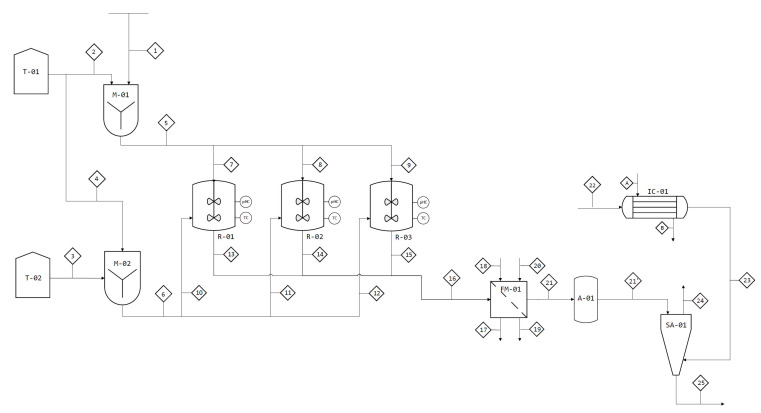
Environmental applications: Full schematic diagram of the plant (flowsheet).

**Figure 6:**
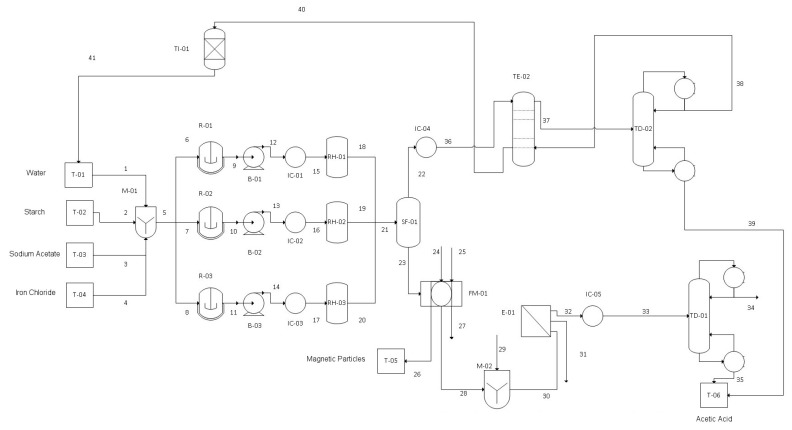
Biotechnological applications: Full schematic diagram of the plant (flowsheet).

In the previous section we determined the operating production capability for both factories. With these numbers defined we have analyzed all the process steps, chosen all the process units and performed all the mass and energy balances required in our plant. Full schematic diagrams are presented in Figure 5 and Figure 6, while in Table 3, Table 4, Table 5, Table 6, Table 7 and Table 8 we present the corresponding nomenclature of the process units and all the mass and heat characteristics of the processing streams.

#### 3.4.1. Raw Materials and Products

In Table 5 and Table 8 are detailed the quantities used of main raw materials, other chemicals, and products/sub-products obtained for both plants.

#### 3.4.2. Operation and Sector Division

(a) Environmental Applications

The magnetic micro and nanoparticle production process will be carried out in three different stages. First, the FeSO_4_·7H_2_O and NH_4_OH solutions will be prepared in two mixers. Secondly, the reaction will be carried out in three batch reactors, and finally, the particles will be washed and dried by means of a magnetic filter and a spray dryer.

The production will be carried out by means of a semicontinuous process due to the need for the reaction to be discontinuous. To ensure continuous supply to the dryer, an accumulator will be used at the outlet of the magnetic filter. The filter and reactors will be synchronized for periods of two hours (the first 40 min represent start-up).

• Preparation of Solutions

Two solutions are to be prepared in two different mixers: FeSO_4_ and NH_4_OH. The first will be prepared with solid FeSO_4_∙7H_2_O and distilled water in a mixer to obtain a concentration equal to 0.2 M. The second mixture is prepared with a NH_4_OH solution that has 28–39% NH_3_ and distilled water. The aim of this mixture is to achieve a pH of 13 and to supply the base needed for the reaction. Both solutions are obtained with constant mechanical agitation. At the end, currents flow from the mixers to the reactors.

• Reaction

Once the solutions are prepared, the reaction is carried out in batch reactors at the optimal operating conditions that have been determined experimentally [28]. The reactor consists of a tank with a perforated plate at the top that aims to distribute the FeSO_4_∙7H_2_O solution from the M-01 mixer so that this solution falls in the form of drops during 10 min (experimental reaction conditions). Then redox reaction occurs, in which iron is oxidized and oxygen is reduced. This reaction will be carried out at room temperature (25 °C) and at atmospheric pressure with constant mechanical stirring for 30 min and at a basic pH close to 13. The conversion of FeSO4 will be 100%. At the end of this time, the reaction mixture obtained will be discharged onto the magnetic filter for a processing time of 10 min. In order to have a semicontinuous process, three equal reactors (R-01, R-02 and R-03) will be used with a time difference of 20 min between their operations. The reaction mixture must be continuously stirred by a mechanical system and must have a temperature and pH control system to ensure that the reaction occurs under optimal conditions. For temperature control, the reactors have an internal coil through which water will circulate at an automatically controlled flow.

• Filtration

The reaction mixture that passes to the washing process will be composed of the obtained magnetite (Fe_3_O_4_) and the other reaction products—(NH_4_)_2_SO_4_—and the aqueous medium that has not reacted. To separate only the magnetite particles from this mixture, a FM-01 magnetic filter will be used, thus the reaction mixture leaving the reactors passes to a magnetic filter. This magnetic filter or high gradient magnetic filter (HGMF)-consists of a ferromagnetic wool surrounded by an electromagnetic coil that when activated generates magnetic field gradients between the fibers of the wool. Hence, as the micro and nanoparticles pass through the filter they are attracted to the wool and are retained by it while the rest of the reaction mixture passes. To eliminate the impurities that may remain on the surface of the particles, a wash is carried out in the same filter and finally the coil is deactivated and a new stream of distilled water is passed in the opposite direction to the one that has been used previously. A total humidity of 30% is obtained due to the water entering the filter. Since the filtering operation is discontinuous, an accumulator will be used at the filter outlet to provide a continuous current to the next processing unit (spray dryer). All currents involved in the above process will be at 25 °C and 1 atm.

• Drying

In the spray dryer, the current coming from the accumulator will enter and will be dried in countercurrent with a hot air stream, corresponding to atmospheric air previously heated up to 90 °C in a heat exchanger (using saturated steam at 250 °C). The dryer has a rotary atomizer and a conical drying chamber. The air taken from the atmosphere will have an average relative humidity of 80%. The humidified air will come out at 40 °C. Then, the magnetite micro and nanoparticles will come out of the drying chamber at 30 °C and 3% humidity. Finally, the obtained magnetic particles are packaged.

• Secondary Operations

In addition to the main line of the process, a series of secondary operations are carried out: the preparation of the solution for pH control in the reactor, the cleaning of the perforated plate of the reactors when the deposition of solute on the plate causes the decrease of the flow and a temperature and pH control system in the reactors.

(b) Biotechnological Applications

The process for the production of nanomagnetic particles (NMPs) can be divided into four stages: mixing, hydrothermal reaction and treatment, separation and washing of NMPs allied with the separation and purification of by-products, and finally the storage of raw materials and products. The plant will operate semicontinuously since the reactions must be carried out batchwise. To do this, we will work in three equal parallel stages, working with a seven-hour lag between them.

• Mixing, Reaction, and Hydrothermal Treatment

The raw materials are first mixed in the appropriate proportions to be subsequently fed to the mixing reactor where reaction (2) occurs. Sodium acetate and chloride are introduced with a mass ratio of 2.74, deionized water at a ratio of 44.7 L/ kg chloride, and starch with a mass ratio of 1.12 with respect to chloride. These reagents are mixed with vigorous stirring in the reactor for 20 min at 80 °C, until producing a homogeneous solution. The reaction conversion may be assumed to be 100% for the chloride.

After finishing the first reaction, the obtained solution is compressed to 2 MPa, heated to the reactor operating temperature and brought to the hydrothermal reactor, where the hydrothermal treatment is carried out for 20 h at 200 °C and reactions (3) and (4) occur. Dissolved oxygen partially oxidizes iron hydroxide to form goethite until a suitable ratio of the two compounds is reached in order to form magnetite. The reaction conversion may be assumed to be 100% for the hydroxide.

• Product Separation

The products subsequently expand to atmospheric pressure in a flash, where separation of acetic acid (AcH) and water from the other components occurs. The gas stream with acetic acid and water is cooled and taken to a liquid-liquid extraction tower where they contact with methyl tert-butyl ether (MTBE), since the percentage of acetic acid in the stream is very small, which makes LL-extraction a cheaper option. The acetic acid exits with the extract, and is subsequently separated with a distillation tower, while the raffinate is taken to an ion exchange tower where pure water is obtained and returned to the storage tank. On the other hand, the liquid current that exits the flash contains the magnetite, the acetate and the salt, and is introduced in the magnetic filter in order to achieve a separation of the magnetite from the rest of the components.

To separate the nanomagnetic particles from the solution, a high gradient magnetic filter is used since it is the most efficient to separate this type of particles. This filter consists of a metallic wool matrix located inside an electromagnetic system, which when activated generates a large magnetic field that attracts the particles to the metallic wool and lets the rest of the solution pass. The solution is passed several times through the filter to ensure that all NMPs are separated. The filter is able to trap 100% of the magnetite particles.

• Flushing and Storage of NMPs

The NMPs, once trapped in the metallic wool, are subjected to a sequential washing with ethanol and water in the same filter to eliminate any impurities that they may have. The wash is performed three times, and each wash uses an amount of each component equal to half the amount of NMPs trapped in the filter. Once the washing process has been carried out, the magnetic field of the magnetic filter is deactivated and the NMPs are separated from the metallic wool by passing a stream of water, being finally stored.

• Separation, Purification and Storage of Acetic Acid

The separated solution of the NMPs containing water, acetic acid, NaCl and CH_3_COONa (NaAc) are taken to a mixer where hydrochloric acid is added to convert all the NaAc into acetic acid, since the separation of NaAc from NaCl is not feasible by other methods. The reaction (7) that occurs is as follows:

CH_3_COONa + HCl→CH_3_COOH + NaCl (7)

where a 1:1 molar ratio will be used so that the reaction shifts completely to the right.

The final stream containing acetic acid, water and NaCl is brought to an evaporator to separate the components. On one hand, the salt will be obtained (on an aqueous form), which will be stored for possible sale, and on the other hand, the gaseous stream of acetic acid and water will be taken to a distillation tower to purify the acetic acid, which will be combined with that obtained in the LL-extraction process, to be stored.

### 3.5. Economic Impact and Profitability

After having determined the maximum treatment capability, the overall process engineering and characterized the different streams, we finally may compute the full economic impact and profitability of the plant.

In Table 9 and Table 10 are indicated the overall costs. In Supplementary Material details are given about the calculation of these values. It is important to notice that many of the sub-totals are calculated based on estimates and, thus, the total numbers are always estimates.

At the end we get a maximum net benefit of 4,214,741 €/year, a net profitability of 34.2%, and a minimum recovery time of 2.92 years for the environmental applications case, and a maximum net benefit of 22,318,031 €/year, a net profitability of 58.0%, and a minimum recovery time of 1.73 years for the biotechnological applications case.

## 4. Discussion

Each intended application of the particles determines the characteristics of the obtained product that reflects on the final engineering process options and manufacturing process. In fact, for environmental applications the final nanomagnetic particles will possess a large polidispersity (even mixed-size: micro- and nanosized particles) and are low-cost bare particles in dry media, while for biotechnological applications the final product must present the desired monodispersity, a surface layer ready for functionalization being highly-priced particles in liquid media. The manufacturing processes are very different: particles are obtained by the reverse co-precipitation method for the environmental case while hydrothermal method is used in the biotechnological case. The production rate is about 15 times higher for the environmental applications plant. The difference in the final product also justifies that the biotechnological plant requires more process steps than the environmental applications plant. Nonetheless, both plants work in a semicontinuous way and both present the step of magnetic filtering for the recovery of the magnetic particles after the reaction stage. The biotechnological plant will be larger than the environmental plant, and the size and cost of the former will be higher than the latter (the total invested capital is almost three times higher); nonetheless profitability is expected to be higher in the biotechnological case (58% opposing to 34.2% in the environmental plant). The maximum net benefit in the biotechnological plant is also more than five times higher than in the environmental case. Anyway, it is important to notice that due to a much higher-level of competition with other companies, the biotechnological applications plant represents a riskier investment.

## 5. Conclusions and Future Perspectives

We have fully designed and studied the industrial implementation of two plants to produce micron and nanomagnetic particles for two different applications (environmental and biotechnological). Both have proven to be viable economically and technically. Full process engineering, including energy and mass balances, was also conducted for both plants. Since the profit is larger than regular bank benefits, investors may consider attractive the construction and startup of any of the designed industrial plants. Additionally, it is expected that this work will serve as the basis for other similar studies for different products, showing the connection between science and practical applications, and to allow scientific improvement of processes bearing in mind their possible industrial implementation.

## Figures and Tables

**Table 1:** Comparison between preselected manufacturing processes ([23,24,25,26]).

Manufacturing Method	Synthesis	Reaction Temp. (°C)	Reaction time	Solvent	Surface-Capping Agents	Size Distribution	Shape Control	Yield
Co-precipitation	Very simple	20–90	Minutes	Water	During/after reaction	Relatively narrow	Not good	High
Microemulsion	Complicated	20–50	Hours	Organic agents	During reaction	Relatively narrow	Good	Low
Thermal decomposition	Complicated	100–320	Hours-days	Organic agents	During reaction	Very narrow	Very good	High
Hydrothermal	Simple	200–250	Hours-days	Water-ethanol	During reaction	Very narrow	Very good	Medium

**Table 2:** Prices of iron oxide nanomagnetic particles (Skyspring Nanomaterials Inc, 2020, [45]).

Product	Price
Iron oxide nanopowder/nanoparticles (alpha-Fe_2_O_3_, 99%, 20–40 nm)	$98/100 g $262/500 g $380/1000 g
Iron oxide nanopowder/nanoparticles (gamma-Fe_2_O_3_, 99%, 20–40 nm)	$98/100 g $262/500 g $380/1 kg
Iron oxide nanopowder/nanoparticles (Fe_3_O_4_, 98 + %, 20–30 nm)	$98/100 g $262/500 g $340/1 kg

**Table 3:** Environmental applications: Streams.

**Materials**	**Unit**	**1**	**2**	**3**	**4**	**5**	**6**	**7**	**8**	**9**	**10**	**11**	**12**	**13**	**14**
FeSO_4_	kg/h	19.68	-	-	-	19.68	-	6.56	6.56	6.56	-	-	-	-	-
H_2_O	kg/h	16.34	647.86	1297.18	3321.00	664.20	4618.19	221.40	221.40	221.40	1539.40	1539.40	1539.40	1540.17	1540.17
NH_4_OH	kg/h	-	-	1691.72	-	-	1691.72	-	-	-	563.91	563.91	563.91	560.88	560.88
Fe_3_O_4_	kg/h	-	-	-	-	-	-	-	-	-	-	-	-	3.33	3.33
(NH4)_2_SO_4_	kg/h	-	-	-	-	-	-	-	-	-	-	-	-	5.71	5.71
Air	kg/h	-	-	-	-	-	-	-	-	-	-	-	-	-	-
Total	kg/h	36.02	647.86	2988.90	3321.00	683.88	6309.91	227.96	227.96	227.96	2103.30	2103.30	2103.30	2110.09	2110.09
Total	kmol/h	1.04	35.96	120.28	184.35	37.00	304.63	12.33	12.33	12.33	101.54	101.54	101.54	101.56	101.56
T	K	298	298	298	298	298	298	298	298	298	298	298	298	298	298
P	kPa	101.33	101.33	101.33	101.33	101.33	101.33	101.33	101.33	101.33	101.33	101.33	101.33	101.33	101.33
Enthalpy	kJ/h	0	0	0	0	0	0	0	0	0	0	0	0	0	0
-	-	-	-	-	-	-	-	-	-	-	-	-	-	-	-
**Materials**	**Unit**	**15**	**16**	**17**	**18**	**19**	**20**	**21**	**21ª**	**22**	**23**	**24**	**25**	**A**	**B**
FeSO_4_	kg/h	-	-	-	-	-	-	-	-	-	-	-	-	-	-
H_2_O	kg/h	1540.17	4620.52	4620.52	10.00	10.00	4.29	4.29	4.29	3.12	3.12	7.10	0.31	32.14	32.14
NH_4_OH	kg/h	560.88	1682.64	1682.64	-	-	-	-	-	-	-	-	-	-	-
Fe_3_O_4_	kg/h	3.33	10.00		-	-	-	10.00	10.00	-	-	-	10.00	-	-
(NH4)_2_SO_4_	kg/h	5.71	17.12	17.12	-	-	-	-	-	-	-	-	-	-	-
Air	kg/h	-	-	-	-	-	-	-	-	195.27	195.27	195.27	-	-	-
Total	kg/h	2110.09	6330.28	6320.28	10.00	10.00	4.29	14.29	14.29	198.40	198.40	202.38	10.31	32.14	32.14
Total	kmol/h	101.56	304.67	304.63	0.56	0.56	0.24	0.28	0.28	6.92	6.92	7.14	0.06	1.78	1.78
T	K	298	298	298	298	298	298	298	298	298	368	318	308	523	523
P	kPa	101.33	101.33	101.33	101.33	101.33	101.33	101.33	101.33	101.33	101.33	101.33	101.33	101.33	101.33
Enthalpy	kJ/h	0	0	0	0	0	0	0	0	0	20,271.67	20,233.54	38.13	55,152.57	34,880.90

**Table 4:** Nomenclature of the processing units in Figure 5, environmental applications.

T-01	Storage Tank 1	T-02	Storage Tank 2
M-01	Mixer 1	M-02	Mixer 2
R-01	Reactor 1	R-02	Reactor 2
R-03	Reactor 3	FM-01	Magnetic Filter
IC-01	Heat Exchanger 1	A-01	Roller Crusher
SA-01	Dryer Atomizer	-	-

**Table 5:** Quantities of raw materials, products, and sub-products per hour, environmental applications.

Materials	Quantit y (kg/h)	Quality
FeSO_4_	19.68	Raw Material
H_2_O	4620.52	Sub-Product/Raw Material
NH_4_OH	1691.72	Raw Material
Fe_3_O_4_	10.00	Raw Material
(NH_4_)_2_SO_4_	17.12	Sub-Product

**Table 6:** Biotechnological applications: Streams.

**Materials**	**1**	**2**	**3**	**4**	**5**	**6**	**7**	**8**	**9**	**10**	**11**	**12**	**13**	**14**	**15**	**16**	**17**	**18**	**19**	**20**	**-**
FeCl_2_·4H_2_O	48.64	0.00	0.00	0.00	48.64	16.21	16.21	16.21	0.00	0.00	0.00	0.00	0.00	0.00	0.00	0.00	0.00	0.00	0.00	0.00	-
CH_3_COONa	0.00	133.26	0.00	0.00	133.26	44.42	44.42	44.42	30.99	30.99	30.99	30.99	30.99	30.99	30.99	30.99	30.99	30.99	30.99	30.99	-
Starch	0.00	0.00	54.47	0.00	54.47	18.16	18.16	18.16	18.16	18.16	18.16	18.16	18.16	18.16	18.16	18.16	18.16	0.00	0.00	0.00	-
H_2_O	0.00	0.00	0.00	2174.99	2174.99	725.00	725.00	725.00	727.95	727.95	727.95	727.95	727.95	727.95	727.95	727.95	727.95	729.40	729.40	729.40	-
CH_3_COOH	0.00	0.00	0.00	0.00	0.00	0.00	0.00	0.00	9.83	9.83	9.83	9.83	9.83	9.83	9.83	9.83	9.83	9.83	9.83	9.83	-
Fe(OH)_2_	0.00	0.00	0.00	0.00	0.00	0.00	0.00	0.00	7.37	7.37	7.37	7.37	7.37	7.37	7.37	7.37	7.37	0.00	0.00	0.00	-
NaCl	0.00	0.00	0.00	0.00	0.00	0.00	0.00	0.00	9.50	9.50	9.50	9.50	9.50	9.50	9.50	9.50	9.50	9.50	9.50	9.50	-
HCl	0.00	0.00	0.00	0.00	0.00	0.00	0.00	0.00	0.00	0.00	0.00	0.00	0.00	0.00	0.00	0.00	0.00	0.00	0.00	0.00	-
C_2_H_5_OH	0.00	0.00	0.00	0.00	0.00	0.00	0.00	0.00	0.00	0.00	0.00	0.00	0.00	0.00	0.00	0.00	0.00	0.00	0.00	0.00	-
MTBE	0.00	0.00	0.00	0.00	0.00	0.00	0.00	0.00	0.00	0.00	0.00	0.00	0.00	0.00	0.00	0.00	0.00	0.00	0.00	0.00	-
Magnetite	0.00	0.00	0.00	0.00	0.00	0.00	0.00	0.00	0.00	0.00	0.00	0.00	0.00	0.00	0.00	0.00	0.00	6.27	6.27	6.27	-
Total (kg/day)	48.64	133.26	54.47	2174.99	2411.36	803.79	803.79	803.79	803.79	803.79	803.79	803.79	803.79	803.79	803.79	803.79	803.79	785.99	785.99	785.99	-
Total (kmol/day)	0.246	1.625	0.336	120.833	123.040	41.01	41.01	41.01	41.34	41.34	41.34	41.34	41.34	41.34	41.34	41.34	41.34	41.25	41.25	41.25	-
Enthalpy (kJ/day)	0.00	0.00	0.00	0.00	25,278.33	8426.11	8426.11	8426.11	17,2967.67	17,2967.67	17,2967.67	173,807.35	173,807.35	173,807.35	550,215.21	550,215.21	550,215.21	547,316.72	547,316.72	547,316.72	-
T(K)	298.00	298.00	298.00	298.00	300.70	300.70	300.70	300.70	353.00	353.00	353.00	353.35	353.35	353.35	473.00	473.00	473.00	473.00	473.00	473.00	-
P(MPa)	0.10	0.10	0.10	0.10	0.10	0.10	0.10	0.10	0.10	0.10	0.10	2.00	2.00	2.00	2.00	2.00	2.00	2.00	2.00	2.00	-
-	-	-	-	-	-	-	-	-	-	-	-	-	-	-	-	-	-	-	-	-	-
-	-	-	-	-	-	-	-	-	-	-	-	-	-	-	-	-	-	-	-	-	-
-	-	-	-	-	-	-	-	-	-	-	-	-	-	-	-	-	-	-	-	-	-
-	-	-	-	-	-	-	-	-	-	-	-	-	-	-	-	-	-	-	-	-	-
-	-	-	-	-	-	-	-	-	-	-	-	-	-	-	-	-	-	-	-	-	-
**Materials**	**21**	**22**	**23**	**24**	**25**	**26**	**27**	**28**	**29**	**30**	**31**	**32**	**33**	**34**	**35**	**36**	**37**	**38**	**39**	**40**	**41**
FeCl_2_·4H_2_O	0.00	0.00	0.00	0.00	0.00	0.00	0.00	0.00	0.00	0.00	0.00	0.00	0.00	0.00	0.00	0.00	0.00	0.00	0.00	0.00	0.00
CH_3_COONa	92.98	0.00	92.98	0.00	0.00	0.00	0.00	92.98	0.00	0.00	0.00	0.00	0.00	0.00	0.00	0.00	0.00	0.00	0.00	0.00	0.00
Starch	0.00	0.00	0.00	0.00	0.00	0.00	0.00	0.00	0.00	0.00	0.00	0.00	0.00	0.00	0.00	0.00	0.00	0.00	0.00	0.00	0.00
H_2_O	2188.21	2186.22	142.27	28.21	0.00	0.00	28.21	142.27	73.92	216.19	0.00	216.19	216.19	215.76	0.43	2186.22	21.86	21.86	0.04	2164.35	2164.35
CH_3_COOH	29.48	29.42	0.05	0.00	0.00	0.00	0.00	0.05	-	68.09	0.00	68.09	68.09	0.14	67.9	29.42	28.35	0.06	28.29	1.07	-
Fe(OH)_2_	0.00	0.00	0.00	0.00	0.00	0.00	0.00	0.00	0.00	0.00	0.00	0.00	0.00	0.00	0.00	0.00	0.00	0.00	0.00	0.00	0.00
NaCl	28.49	0.00	28.49		0.00	0.00	0.00	28.49	0.00	94.26	94.26	0.00	0.00	0.00	0.00	0.00	0.00	0.00	0.00	0.00	0.00
HCl	0.00	0.00	0.00	0.00	0.00	0.00	0.00	0.00	40.82	0.00	0.00	0.00	0.00	0.00	0.00	0.00	0.00	0.00	0.00	0.00	0.00
C_2_H_5_OH	0.00	0.00	0.00	0.00	0.00	0.00	0.00	0.00	0.00	0.00	0.00	0.00	0.00	0.00	0.00	0.00	886.26	886.26	0.00	0.00	0.00
MTBE	0.00	0.00	0.00	0.00	28.21	0.00	28.21	0.00	0.00	0.00	0.00	0.00	0.00	0.00	0.00	0.00	0.00	0.00	0.00	0.00	0.00
Magnetite	18.81	0.00	18.81	0.00	0.00	18.81	0.00	0.00	0.00	0.00	0.00	0.00	0.00	0.00	0.00	0.00	0.00	0.00	0.00	0.00	
Total (kg/day)	2357.97	2215.64	282.61	28.21	28.21	18.81	56.42	263.90	114.74	378.54	94.26	284.28	284.28	215.90	68.38	2215.64	936.47	908.17	28.33	2165.43	2164.35
Total (kmol/day)	123.76	121.95	9.61	1.57	0.61	0.08	2.18	9.53	5.24	14.77	1.63	13.15	13.15	11.99	1.16	121.95	11.74	11.26	0.47	120.26	120.24
Enthalpy (kJ/day)	1,641,950	5,870,529	0.00	0.00	0.00	0.00	0.00	0.00	0.00	−8416.8	7895.9	552,854	−422,597	1,217,950	778,32.1	3,753,140.1	90,649.0	85,258.2	5398.7	390,831.3	0.00
T(K)	473.00	473.00	298.00	298.00	298.00	298.00	298.00	298.00	298.00	278.00	391.00	391.00	374.55	372.88	390.68	353.00	342.57	341.16	390.72	341.16	298.00
P(MPa)	2.00	0.10	0.10	0.10	0.10	0.10	0.10	0.10	0.10	0.10	0.10	0.10	0.10	0.10	0.10	0.10	0.10	0.10	0.10	0.10	0.10

**Table 7:** Nomenclature of the process units in Figure 6, biotechnological applications.

T-01	Storage Tank 1	T-02	Storage Tank 2
T-03	Storage Tank 3	T-04	Storage Tank 4
T-05	Storage Tank 5	T-06	Storage Tank 6
IC-01	Heat Exchanger 1	IC-02	Heat Exchanger 2
IC-03	Heat Exchanger 3	IC-04	Flash Separator 4
IC-05	Heat Exchanger 5	SF-01	Flash Separator 1
M-01	Mixer 1	M-02	Mixer 2
R-01	Reactor 1	R-02	Reactor 2
R-03	Reactor 3	B-01	Pump 1
B-02	Pump 2	B-03	Pump 3
RH-01	Hydrothermal Reactor 1	RH-02	Hydrothermal Reactor 2
RH-03	Hydrothermal Reactor 3	FM-01	Magnetic Filter
E-01	Evaporator 1	TE-01	Extraction Tower 1
TD-01	Distillation Tower 1	TD-02	Distillation Tower 2
TI-01	Ion Exchange Tower 1	-	-

**Table 8:** Quantities of raw materials, products and sub-products per day, environmental applications.

Materials	Quantity (kg/day)	Quality
FeCl_2_·4H_2_O	48.64	Raw Material
Sodium Acetate	133.26	Raw Material
Starch	54.47	Raw Material
Water	2174.99	Raw Material/Sub-product
Magnetite	18.81	Product
NaCl	94.26	Sub-Product
HCl	40.82	Raw Material
Ethanol	886.26	Separation Aid
MTBE	28.21	Separation Aid

**Table 9:** Overall costs of industrial implementation of NMP production for environmental applications.

Cost	€	Invested Capital	€
1.1 Raw Materials	9,302,682	1.1 Instrumentation	876,200
1.2 Direct Human Labor	2,264,731	1.2 Initial Setup	1,180,970
1.3 Indirect Human Labor	503,207	1.3 Piping and Valves	525,720
1.4 General Services	1704	1.4 Measuring and Control	131,430
1.5 Supplies	41,745	1.5 Heat Isolation	35,048
1.6 Conservation Expenses	208,727	1.6 Electrical Installation	131,430
1.7 Laboratory	679,419	1.7 Land and Structures	1,541,610
1.8 Board and Technical Staff	467,565	1.8 Auxiliary Facilities	350,480
1.9 Amortization	73,880	1.9 Project and Design	497,244
1.10 Packaging	6,110,400	1.10 Constructor Hiring	286,373
1.11 Taxes and Insurances	139,151	1.11 Unexpected Expenses	685,117
TOTAL COST OF FABRICATION	19,791,507	1.12 Preliminary Research, Study and Startup	715,933
2.1 Comercial Expenses	3,958,301	TOTAL IMMOBILIZED	6,957,556
2.2 Management	890,618	-	-
2.3 Financial Expenses	49,257	CIRCULATING CAPITAL	5,356,704
2.4 Research	208,727	-	-
2.5 Technical Services	33,935	TOTAL INVESTED CAPITAL	12,314,260
TOTAL COST OF MANAGEMENT	5,140,838	-	-
TOTAL PRODUCTION COSTS	24,932,346	TOTAL INCOME	30,552,000

**Table 10:** Overall costs of industrial implementation of NMP production for biotechnological applications.

Cost	€	Invested Capital	€
1.1 Raw Materials	15,888,945	1.1 Instrumentation	1,714,802
1.2 Direct Human Labor	2,193,938	1.2 Initial Setup	1,668,069
1.3 Indirect Human Labor	602,897	1.3 Piping and Valves	1,028,881
1.4 General Services	9,048,525	1.4 Measuring and Control	514,441
1.5 Supplies	432,988	1.5 Heat Isolation	120,036
1.6 Conservation Expenses	863,263	1.6 Electrical Installation	342,960
1.7 Laboratory	658,181	1.7 Land and Structures	6,257,220
1.8 Board and Technical Staff	507,229	1.8 Auxiliary Facilities	685,921
1.9 Amortization	128,721	1.9 Project and Design	1,284,795
1.10 Taxes and Insurances	577,318	1.10 Constructor Hiring	739,940
TOTAL COST OF FABRICATION	30,902,006	1.11 Unexpected Expenses	2,096,496
2.1 Comercial Expenses	6,180,401	1.12 Preliminary Studies	10,103,064
2.2 Management	479,412	1.13 Preliminary Startup	2,309,272
2.3 Financial Expenses	5,776,170	-	-
2.4 Research	1,154,636	TOTAL IMMOBILIZED	28,865,898
2.5 Technical Services	1,904,636	CIRCULATING CAPITAL	9,641,903
TOTAL COST OF MANAGEMENT	14,340,620	TOTAL INVESTED CAPITAL	38,507,801
TOTAL PRODUCTION COSTS	45,242,625	TOTAL INCOME	75,000,000

## Supplementary Materials

The following are available online at https://www.mdpi.com/1996-1944/13/11/2477/s1, Table S1: Costs of raw materials/income of selling subproducts (environmental applications), Table S2: Costs of raw materials/income of selling subproducts (biotechnology applications), Table S3: Labor costs for the environmental applications case (direct human labor), Table S4: Labor costs for the biotechnology applications case (direct human labor), Table S5: Labor costs for the environmental applications case (indirect human labor), Table S6: Labor costs for the biotechnology applications case (indirect human labor), Table S7: General services of the environmental applications plant, Table S8: Labor costs for the environmental applications case (chief personnel), Table S9: Chief personnel labor costs for the biotechnology applications case, Table S10: Management personnel labor costs for the biotechnology applications case, Table S11: Technical services labor costs for the environmental applications case, Table S12: Fixed immobilized capital (for EA plant), Table S13: Fixed immobilized capital (for BA plant).
