# Supplementary material for: Upscale Design, Process Development, and Economic Analysis of Industrial Plants for Nanomagnetic Particle Production for Environmental and Biomedical Use
Source: Materials (Basel). 2020 May 29;13(11):2477. doi: 10.3390/ma13112477 (PMC7321212; doi:10.3390/ma13112477)
Supplement: Supplementary file 1 [file materials-13-02477-s001.pdf]

materials

Upscale Design, Process Development and Economic Analysis of Industrial Plants for Nanomagnetic Particle Production for Environmental and Biomedical Use

Paulo A Augusto [1,*], Teresa Castelo-Grande [2], Diana Vargas [1], Alvaro Pascual [1], Lorenzo Hernández [1], Angel M Estevez [1] and Domingos Barbosa [2]

[1] Departamento de Ingeniería Química y Textil, Facultad de Ciencias Quimicas, Universidad de Salamanca, Plaza de los Caídos, 1-5, 37008 Salamanca, Spain
[2] LEPABE–Laboratory for Process Engineering, Environment, Biotechnology and Energy, Faculty of Engineering, University of Porto, Rua Dr. Roberto Frias, 4200-465 Porto, Portugal
* Correspondence: pauloaugusto@usal.es

Supplementary material

It is important to notice that it is impossible to know precisely the amount of some of the expenses related to any plant, unless they are already built. Therefore, in the design and planning stage estimations are always the best solutions. There are several scientifically accepted methods in literature which describe the best ways to achieve this estimation, and we have followed then (e.g. [S1] and [S2]). However, it is important to notice that some methods diverge on the approach, and thus we must adopt the method which best suits the case under evaluation: a plant for biotechnology applications (BA) imposes different requisites than a plant for environmental applications (EA). Hence, we have applied for each expense or profit the best estimation considering the type of plant, which sometimes leads to different estimation methods being applied for the same expense or profit section on each of the plants under study.

A-Calculations Regarding the Size of the Plant

(a) Income

- Environmental Applications Plant (EA)

The total income, due to selling the magnetic particles will be:

$$V = q * P$$

$$V\left(€/_{year}\right) = 80400\ \frac{kg}{year} * 380\frac{€}{kg} = 30\ 552\ 000\ €/year$$

where V is the total income, q the maximum capacity of the plant and P the price of the particles

- Biotechnological Applications Plant (BA)

The total income, in this case, is:

$$V\left(€/_{year}\right) = 6300\frac{kg}{year} * 11904.76\frac{€}{kg} = 75\ 000\ 000\ €/year$$

(b) Costs

To determine the first estimate of the costs, we will use the following formula [S1]:

$$C = M_1 + M_5 + 1.5M_2 + 0.3I$$

where C is the total cost, M_1 the raw material costs, M_5 the general services costs, M_2 the man labor costs and I the immobilized costs.

(b1) Raw Materials

- EA

In the defined process the raw materials will be $FeSO_4{\cdot}7H_2O$, NH_4OH and H_2O.

For water we will use 31,909.68 m^3/year which supposes a cost 97.74 €/m^3 (includes price of purification stage), thus the total cost will be 3,118,716 €/year.

In the case of $FeSO_4{\cdot}7H_2O$, this will be supplied at the price of 4,823.41 €/tonne, and as 289,62 tonnes/year are required, the total cost will be 1,396,950 €/year.

The requirements on NH_4OH are 24,030.79 tonnes/year at the price of 199.20 €/tonne, therefore the associated cost will be 4,787,016 €/year.

The total raw materials cost (M_1) will be 9,302,682 €/year.

- BA

In this case the raw materials will be $FeCl_2{\cdot}4H_2O$, CH_3COONa, starch and H_2O.

For water we will use 728,623 liters/year, assuming a cost of about 12.10 €/L (includes price of purification stage–much more demanding than in EA), thus the total cost will be 8,816,338.30 €/year.

In this case, the $FeCl_2{\cdot}4H_2O$ will be supplied at the price of 181 €/kg, and as 16,292 kg/year are needed, this represents a total cost of 2,948,852 €/year.

44,642 kg/year of CH_3COONa are needed, which represent a cost of 2,392,811.20 €/year (53.60 kg/year is the price of acquisition of this raw material).

In what starch is concerned, 18,247.04 kg/year are required, and if we multiply by its price (179 €/kg) we get its cost (3,266,220.16 €/year).

On the other end, we also produce as sub-product acetic acid. The selling price of this reagent is 47 €/kg. As we will produce 32,665.47 kg/year of this subproduct, the total profit on selling acetic acid will be 1,535,277.09 €/year. For practical reasons, we will consider this income in this section, which implies that we decrease this amount in the total raw materials cost.

The total raw materials cost (M_1) will be 15,888,944.60 €/year.

(b2) General Services

In general services we consider costs such as electricity, steam, compressed air, etc. This cost usually is calculated as 10–20% of the total costs. We have assumed 20% ($M_S = 0.2$ C) for both plants.

(b3) Direct Manpower (M_z)

The required manpower will be at this stage evaluated by the Andres method [S1]:

$$\frac{Hh}{Tm * op} = 61.33 * q^{-0.82}$$

where *Hh* is the manpower per hour required, *Tm* is total production (tonne/year), *op* the number of process sections of the plant, and *q* the maximum capacity of the plant (tonne/day).

- EA

The plant will work continuously during the year, 24 hr per day and 7 days per week, 11 months per year. It will have 3 shifts of work, 5 days per week, the total number of process sectors 4.

Therefore, the total number of manpower-hour per year is 64,962 Hh/year, which corresponds to a total cost of M_2 = 1,544,788 €/year and a total number of direct manpower of 24 workers.

- BA

The plant will work semi-continuously during the year, 24 hr per day and 5 days per week, 48 weeks per year. The total number of process sectors is 4.

The total number of manpower-hour per year is 1,591 Hh/year, which corresponds to a total cost of M_2 = 909,748 €/year and a total number of direct manpower of 21 workers.

(b4) Immobilized Capital

The immobilized capital is composed by three different types of costs: the fixed active (I_A), the previous studies (I_B) and the start-up costs (I_C).

$$I = I_A + I_B + I_C$$

- EA

Its total is 39,371,134 €, according to the values of the different types of costs, detailed in the next sections.

- BA

Its total is 135,648,400 €, according to the values of the different types of costs, detailed in the next sections.

(b41) Fixed active costs

This type of costs are the enterprise goods that may not be sell in the short time and that are not destined for sale: e.g. instrumentation, office material, etc. For its estimation we will use the "giro coefficient method" [S1]

$$g = \frac{V}{I_A}$$

where V is the sales income and g a coefficient that changes according to the type of factory. For chemical industries it has the general value of 0.97.

- EA

In this case, I_A is equal to 31,496,907 €.

- BA

In this case, I_A is equal to 77,319,588 €.

(b42)

- EA

The costs associated with the preliminary studies, as we are dealing with an industry of new products and high-production rate, is estimated as being 12% of the total immobilized (I_B = 0.12 I).

- BA

The costs associated with the preliminary studies, as we are dealing with an industry of improved products and low-production rate, is estimated as being 35% of the total immobilized (I_B = 0.35 I).

(b43) Start-up costs

This is a value that also depends on the immobilized, and the fixed active costs. For both cases it will correspond to 8% of the total immobilized capital (I_C = 0.08 I).

(b5) Total Costs

- Environmental Applications (EA)

Applying all the data, which is justified as follows, we may determine the value of the total costs to be 29,289,005 €/year.

- Biotechnological Applications (BA)

Applying all the data, which is justified as follows, we may determine the value of the total costs to be 72,435,108 €/year.

B-Calculations Regarding Economic Impact and Profitability

The majority of estimations are based on details given in [S1] and [S2]

II.1–Production Cost

The total Production cost will be the sum of the Fabrication Cost (M) and the Management Cost (G)

II.1.1–Fabrication Cost

(a) Cost of Raw Materials/Income of Selling Subproducts

- EA

Table S1.–Costs of Raw Materials/Income of Selling Subproducts (Environmental Applications).

Raw Material	Price (€/tonne)	Quantity (tonne/year)	Cost/Income (€/year)
$FeSO_4{\cdot}7H_2O$	4,823.41	289.62	1,396,950 (cost)
NH_4OH	199.20	24,030.79	4,787,016 (cost)
H_2O	97.74	31,909.68	3,118,716 (cost)
**Total: 9,302,682 €/year**			

- BA

Table S2.–Costs of Raw Materials/Income of Selling Subproducts (Biotechnology Applications).

Raw Material	Price (€/kg)	Quantity (kg/year)	Cost/Income (€/year)
**$FeCl_2{\cdot}4H_2O$**	181	16 292	2,948,852 (cost)
**CH_3COONa**	53.60	44 642	2,392,811.20 (cost)
**Starch**	179.00	18,247.04	3,266,220.16 (cost)
**H_2O**	12.10	728,623	8,816,338.30 (cost)
**Acetic Acid**	47.00	32,665.47	1,535,277.09 (income)
**Total: 15 888 944.60 €/year**			

(b) Direct Human Labor

As previously pointed out, the plants will be working continuously or semi-continuously and therefore shifts must be applied in several of the work positions, which implies an extra payment to workers, when working by night.

In Tables S3 and S4 are presented the labor costs for the direct human labor.

Table S3.-Labor costs for the Environmental Applications case (direct human labor).

Job	Number of Workers	Annual Salary (€)	Extra Annual (€)	Social Security (€/year)	Total (€)
Mechanic	10	21,890.98	11,436	6,437	397,639.80
Plumber/Builder	6	21,890.98	11,436	6,437	238,583.90
Electrical Technician	4	21,890.98	11,436	6,437	159,055.90
Laboratory Technician	4	21,890.98	11,436	6,437	397,639.80
Operator Zone 1	3	19,211.04	11,436	5,080	1,071,811.20
Total = **2,264,730.60 €/year**					

Table S4.–Labor costs for the Biotechnology Applications case (direct human labor).

Job	Number of Workers	Annual Salary (€)	Extra Annual (€)	Social Security (€/year)	Total (€)
Mechanic	7	26,911.50	15,667.90	7,236.50	348,711.70
Plumber/Builder	7	26,911.50	15,667.90	7,236.50	348,711.70
Electrical Technician	7	26,911.50	15,667.90	7,236.50	348,711.70
Laboratory Technician	22	18,149.60	11,893.70	5,710.70	786,587.10
Operator Zone 1	6	32,700.50	19,034.70	8,467.50	361,216.10
Total = **2,193,938.15 €/year**					

(c) Indirect Human Labor

In Tables S5 and S6 are presented the labor costs for the indirect human labor.

Table S5.–Labor costs for the Environmental Applications case (indirect human labor).

Job	Number of Workers	Annual Salary (€)	Extra Annual (€)	Social Security (€/year)	Total (€)
Cleaning Personnel	3	14,892.40	10,038	4,379	87,928.10
Security	5	19,211.00	10,038	4,686	169,675.20
Commercial	1	19,211.00	10,038	4,686	33,935.00
Receptionist	2	19,211.00	10,038	4,686	67,870.10
Maintenance	1	19,211.00	10,038	4,686	33,935.00
Nurse	2	31,124.80	16,275	7,532	109,863.60
Total = **503,207.10 €/year**					

Table S6.–Labor costs for the Biotechnology Applications case (indirect human labor)

Job	Number of Workers	Annual Salary (€)	Extra Annual (€)	Social Security (€/year)	Total (€)
Cleaning Personnel	3	15,646.20	9,094.80	4,379.00	87,360.00
Security	2	18,149.60	11,893.70	5,710.60	71,507.90
Commercial	2	18,149.60	11,893.70	5,710.60	71,507.90
Receptionist	1	18,149.60	11,893.70	5,710.60	35,754.00
Maintenance	1	18,149.60	11,893.70	5,710.60	35,754.00
Nurse	5	32,700.50	19,034.70	8,467.50	301,013.40
Total = **602,897.20 €/year**					

(d) General Services

In this section are included costs such as refrigeration, etc. In the case of Environmental Applications, they are easier to compute and are presented in Table S7. For Biotechnological Applications we are able to estimate this cost to be 20% of the total production costs, which makes a total of 9,048,525 €/year (BA).

Table S7.–General Services of the Environmental Applications Plant.

Service	kg/h	€/kg	h	€
Superheated Steam	32	0.00664	7,920	1,682.80
Refrigerated Water	1	0.00266	7,920	21.10
**Total** 1,703.90 €/year				

(e) Supplies

In this section are considered the items that are not included in the manufacturing process, mainly disposable material, etc. It is usually estimated to be valuing between 0.5% and 2% of the total immobilized, depending on the complexity of the plant. In this case it is supposed to be 0.6% for EA and 1.5% for BA, which corresponds to 41,745.34 €/year and 432,988.47 €/year, respectively.

(f) Maintenance

In this section are included the periodic maintenance of instrumentation and equipment. For chemical-environmental applications we may assume a lower estimate as corresponding to 3% of the total Immobilized, hence, 208,726.68 €/year. For biotechnological industries it usually corresponds to 6% of the fixed capital, and thus, in this case, 863,263.18 €/year.

(g) Laboratory

For the laboratory work is usually assumed 30% of the direct human labor, thus, in this case it represents 679,419.18 € /year for EA and 658,181.45 € /year for BA.

(h) Chief Personnel

In Tables S8 and S9 are presented the chief personnel costs.

Table S8.–Labor costs for the Environmental Applications case (chief personnel)

**Job**	**Number of Workers**	**Annual Salary (€)**	**Extra Annual (€)**	**Social Security (€/year)**	**Total (€)**
IC Technician	5	31,124.80	16,275	9,152	282,759.10
Manager	1	31,124.80	16,275	9,152	56,551.80
Process Engineer	1	31,124.80	16,275	9,152	56,551.80
Managing Director	1	39,464.50	20,634	11,604	71,702.50
Total = **467,565.20 €/year**					

Table S9.–Chief Personnel labor costs for the Biotechnology Applications case

Job	Number of Workers	Annual Salary (€)	Extra Annual (€)	Social Security (€/year)	Total (€)
Managing Director	1	41,462.40	24,134.80	13,045.80	78,643.00
Process Engineering	1	31,902.90	19,034.70	10,289.00	61,226.60
Environmental and Safety Engineering	1	31,902.90	19,034.70	10,289.00	61,226.60
IC Technician	5	31,902.90	19,034.70	10,289.00	306,132.90
Total = **507,229.10 €/year**					

(i) Amortization

This section takes into account the life span of the equipment. We take as life span the usual figures concerning each type of equipment. Therefore, this cost is estimated as 128,720.60 €/year (EA) and 73,880.00 €/year (BA).

(j) Taxes

This section takes into consideration taxes related to the chemical plant (general worldwide), not including the taxes due to profits. It usually is estimated as 1% of the Immobilized. Hence, in our case is estimated to be 69,575.60 €/year (EA) and 288,659.00 €/year (BA).

(k) Insurance

In this section we consider the insurance of the plant (equipment, facilities, etc.). It is usually 1% of the Immobilized, so, in our case it is estimated to represent a cost of 69,575.60 €/year (EA) and 288,659.00 €/year (BA).

(l) Total Cost of Fabrication

TOTAL FABRICATION COSTS: 19,791,507.20 €/year (EA) and 30,902,005.70 €/year (BA).

II.1.2–Management Costs

(a) Commercial Costs

The commercial costs are estimated to be 20% of the total fabrication costs, thus representing a total value of 3,958,301.40 €/year (EA) and 6,180,401.10 €/year (BA).

(b) Financial Costs

In this item we consider the amount of money that could be earned, if instead of investing it in each plant, it would be invested in a fixed deposit in a bank or other competing alternatives. For Environmental Applications we consider the medium tax value (of the banks) of 0.4%, and thus we get a total cost of 49,257.00€/year. For Biotechnological Applications several alternative opportunities are usually available, therefore, we will assume a very conservative value of 15%, and thus we get a total cost of 5,776,170.20 €/year.

(c) Management

In this section we consider the administrative management costs, or alternatively (depending on the type and dimensions of the plant) the cost of a full sector of the plant dedicated to management. For Environmental Applications it is estimated to be 4.5% of the total fabrication costs, therefore giving a value of 890,617.80 €/year. For Biotechnological Applications a simpler sector of

management is required, therefore in Table S10 are presented the costs related to this sector that give a total of 479,412.30 €/year.

Table S10.–Management Personnel labor costs for the Biotechnology Applications case

Job	Number of Workers	Annual Salary (€)	Extra Annual (€)	Social Security (€/year)	Total (€)
**Administrative**	5	22,999.21	10,557.74	7,236.54	203,967.45
**Recepcionist**	1	18,149.58	9,746.12	5,710.64	33,606.34
**Commercial**	2	18,149.58	9,746.12	5,710.64	67,212.68
**Secretary**	2	18,149.58	9,746.12	5,710.64	67,212.68
**Manager**	1	32,700.52	15,667.94	10,288.98	58,657.44
**Accountant**	1	26,911.47	13,376.70	8,467.50	48,755.67
Total = **479,412.30 €/year**					

(d) Research, Development and Technical Services

The costs on R&D may be estimated as 3–4% of the Immobilized, depending on the level of novelty of the product. For Environmental Applications 3% is more appropriate, and thus the R&D costs for this type of plant is 208,726.70 €/year. For Biotechnological Applications, and due to the higher novelty, we define 4% as more appropriate, and thus the R&D costs for this type of plant is 1 154,635.90 €/year. Considering Technical Services for the Environmental Applications, the hiring of a person is considered enough, like detailed in Table S11 (Total Costs 33,935.40 €/year), while for Biotechnological Applications the costs of technical services must be calculated as 10% of the total selling income (Total cost of 750,000 €/year).

Table S11.–Technical services labor costs for the Environmental Applications case

Job	Number of Workers	Annual Salary (€)	Extra Annual (€)	Social Security (€/year)	Total (€)
**Administrative**	1	19,211.44	10,038	4,686	33,935.44
Total = **33,935.40 €/year**					

(e) Total Cost of Management

TOTAL COST OF MANAGEMENT: 5 140,838.40 €/year (EA) and 14 340,619.50 €/year (BA).

II.1.3–Total Cost of Production

Total Cost of Production = Total Cost of Fabrication (II.1.1) + Total Cost of Management (II.1.2), thus:

TOTAL COST OF PRODUCTION: 24,932,345.60 €/year (EA) and 45,242,625.20 €/year (BA).

II.2–Invested Capital

The Invested Capital is the sum of two parts: the immobilized and the working capital

II.2.1–Fixed Immobilized Capital

This is the amount of invested money that will not be recovered, and is calculated as the sum of parts (a) to (h) that are detailed in what follows. They give a total of 4,772,888 €/year (EA) and 12 332,331.20 €/year (BA)

(a) Equipment and Instrumentation

For Environmental Applications the total cost of equipment is 876,200 €/year while for Biotechnological Applications it reaches the 1,714,802.20 €/year.

(b) Assembly and Start-up

Usually the costs for assembly and startup of the devices and equipment are determined as a percentage of the total equipment cost, therefore will be a total of 1,180,970 €/year (EA) and 1,668,069 €/year (BA).

(c) Tubing and valves

The cost of tubing and valves is estimated as 60% of the total equipment costs, and hence will be a total of 525,720 €/year (EA) and 1,028,881.30 €/year (BA).

(d) Measuring and Control Devices

Costs of this section are usually estimated between 15–30% of the total equipment costs, depending on the level of control required for the plant. For Environmental Applications we consider to be 15% the appropriate value, while for Biotechnological Applications is more appropriate to consider 30%, and therefore the total costs are 131,430 €/year (EA) and 514,440.70 €/year (BA).

(e) Thermal Isolation

The cost for the thermal isolation will be considered to be 4% of the total equipment costs in the case of Environmental Applications (35,048 €/year) and 7% of the total equipment costs in the case of Biotechnological Applications (120,036.20 €/year) as thermal isolation in BA is superior to EA (higher temperatures must be maintained and operated).

(f) Electrical Installation

The cost for the electrical installation~~,~~ usually corresponds to a percentage of the total equipment costs. For the Environmental Applications we calculate it as 15% of the equipment cost (131,430 €/year), while for Biotechnological Applications it is calculated as 20% of the equipment cost, as more equipment and area is covered (342,960.40 €/year).

(g) Land Property and Buildings

This is calculated by adding the cost of the land as a percentage of the total equipment costs. For the Environmental Applications Plant it is assumed 5% of the equipment costs, that added to the cost of the land gives a total of 1,541,610 €/year. For the Biotechnological Applications Plant it is assumed 15% of the equipment costs, that added to the cost of the land gives a total of 6,257,220.30 €/year.

(h) Auxiliary Facilities

For this type of cost, usually it is estimated a value of 40% of the total equipment cost, that gives a total value of 350,480 €/year (EA) and 685,920.90 €/year (BA).

II.2.2–Other Costs

(a) Design and Engineering

This part contemplates all the design and engineering works done for the construction of the plant. Usually it is estimated as about 10.4% (including VAT) of the total Fixed Capital, and therefore will be a total of 497,244.20 €/year (EA) and 1,284,794.60 €/year (BA).

(b) Contract of Works

It is supposed to be 6% of the fixed capital, thus will be a total of 286,373.30 €/year (EA) and 739,939.90 €/year (BA).

(c) Contingency

In this section are included all the unexpected extra costs that may appear. It is usually estimated as being a percentage of the Fixed Capital, depending on the level of estimative costs we have made and the unexpected problems we foresee to be handling. For the case of Environmental Applications about 32.35% are considered (including VAT) (total of 685,117.20 €/year) and 17% for the case of Biotechnological Applications (total of 2,096,496.30 €/year).

(d) Preliminary Research, Studies and Startup

In the case of Environmental Applications, the costs related to this section are calculated as 15% of the Fixed Capital (total 715,933.20 €/year). For the case of Biotechnological Applications, they are computed as 43% of the total Immobilized (total 12,412,336.20 €/year).

Fixed Immobilized Capital

In tables S12 and S13 we present a sum-up of all the parts of the calculated total Fixed Immobilized Capital for the two types of Plants.

Table 12.–Fixed Immobilized Capital (for EA plant).

		**Type of Cost**	**Cost (€)**
**Immobilized Capital**	**Fixed Capital: P.E.M**	Equipment and Instrumentation	876,200
		Assembly and Start-up	1,180,970
		Tubing and valves	525,720
		Measuring and Control Devices	131,430
		Thermal Isolation	35,048
		Electrical Installation	131,430
		Land property and buildings	1,541,610
		Auxiliary facilities	350,480
	**Total Cost of the Fixed Capital**		4,772,888
	Design and Engineering		497,244.20
	Contract of Works		286,373.30
	Contingency		685,117.20
	Preliminary Research and Studies and Startup		715,933.20
	**Total of Immobilized Cost**		6,957,555.90

Table S13.–Fixed Immobilized Capital (for BA plant).

		Type of Cost	Cost (€)
**Immobilized Capital**	**Fixed Capital: P.E.M**	Equipment and Instrumentation	1,714,802.20
		Assembly and Start-up	1,668,069.20
		Tubing and valves	1,028,881.30
		Measuring and Control Devices	514,440.70
		Thermal Isolation	120,036.20
		Electrical Installation	342,960.40
		Land property and buildings	6,257,220.30
		Auxiliary facilities	685,920.90
	**Total Cost of the Fixed Capital**		12,332,331.20
	Design and Engineering		1,284,794.60
	Contract of Works		739,939.90
	Contingency		2,096,496.30
	Preliminary Research and Studies and Startup		12,412,33.20
	**Total of Immobilized Cost**		28,865,898.10

II.3–Working Capital

This is the part of the Capital that may be recovered.

II.3.1–Raw Materials and Auxiliaries

To compute this cost we may apply

$$M'_1 * \left(\frac{q}{12}\right)$$

where M'_1 is the cost of raw materials by unit of product and q the annual production quantity. This gives a final cost of 775,223.50 €/year (EA) and 1,324,078.70 €/year (BA) for this section of the costs.

II.3.2–Materials in Manufacturing

This cost may be computed with the knowledge of the manufacturing cycle. In the case of the Environmental Applications Plant corresponds to 0.012 months and therefore the associated costs will be 9,895.80 €/year, while for the Biotechnological Applications Plant we assume 0.033 months and an associated cost of 42,490.30 €/year.

II.3.3–Reserve of the Manufactured Product in the Warehouse

The costs corresponding to this section are computed as 1,649,292.30 €/year (EA) and 2,575,167.10 €/year (BA), as they are obtained as the fabrication cost per month.

II.3.4–Sales Pending Collection

Corresponds to the credit that is given to buyers. In our case it corresponds to 1,273,000 €/year (EA) and 3,125,000 €/year (BA)–calculated as the income (of the selling of the product) per each 15 days.

II.3.5–Available in ATM's and Banks for Immediate Payments

It corresponds to 1,649,292.30 €/year (EA) and 2,575,167.10 €/year (BA). They are estimated in a similar way as the reserve of the manufactured product in the warehouse.

II.3.6–Calculation of Working Capital

TOTAL WORKING CAPITAL: 5,356,703.80 €/year (EA) and 9,641,903.30 €/year (BA)

II.4–Total Invested Capital

TOTAL INVESTED CAPITAL = WORKING CAPITAL + IMMOBILIZED
TOTAL INVESTED CAPITAL: 12,314,259.70 €/year (EA) and 38,507,801.40 €/year (BA)

II.5–Total Sell Income

This was already calculated previously and is equal to 30,552,000 €/year (EA) and 75,000,000 €/year (BA).

II.6–Profitability

II.6.1–Gross Annual Profit

This is computed as the difference between Costs and Sells, and in this case gives a value of 5,619,654.40 €/year (EA) and 29,757,374.80 €/year (BA).

II.6.2–Gross Annual Profitability

Is computed as the Gross Annual Profit divided by the Invested Capital. In our case it gives a value of 45.6% (EA) and 77.3% (BA).

II.6.3–Net Annual Profit

Is equal to the Gross Annual Profit minus the Taxes. In our case as the Society Tax is 25% in Spain, we get a total Net Annual Profit of 4,214,740.80 €/year (EA) and 22,318,031.10 €/year (BA).

II.6.4–Net Annual Profitability

In our case it gives 34.2% (EA) and 58.0% (BA)

References:

[S1]–Vian-Ortuño Á. El pronóstico económico en química industrial. EUDEMA, S. A., Madrid, Spain, 1991.
[S2]–Sinnot R. Diseño en Ingeniería Química. Reverté, Barcelona, Spain, 2012.

End of supplementary material.
